# Mutations in *ATP6V1B1 and ATP6V0A4* genes cause recessive distal renal tubular acidosis in Mexican families

**DOI:** 10.1002/mgg3.205

**Published:** 2016-02-14

**Authors:** Laura I. Escobar, Christopher Simian, Cyrielle Treard, Donia Hayek, Carolina Salvador, Norma Guerra, Mario Matos, Mara Medeiros, Sandra Enciso, María Dolores Camargo, Rosa Vargas‐Poussou

**Affiliations:** ^1^ Departamento de Fisiología Facultad de MedicinaUniversidad Nacional Autónoma de MéxicoMexico CityMexico; ^2^ Département de GénetiqueHôpital Européen Georges PompidouParisFrance; ^3^ Servicio de NefrologíaHospital General del Centro Médico Nacional La Raza, Instituto Mexicano del Seguro Social (IMSS)Mexico CityMexico; ^4^ Laboratorio de Investigación en NefrologíaHospital Infantil de México Federico GómezMexico CityMexico; ^5^ UMAE Hospital de EspecialidadesCentro Médico Nacional del Noreste, IMSSNo. 25.MonterreyMexico

**Keywords:** Hearing loss, hypokalemia, nephrocalcinosis, renal tubular acidosis

## Abstract

**Background:**

Autosomal recessive distal renal tubular acidosis (dRTA) is a rare disease characterized by a hyperchloremic metabolic acidosis with normal anion gap, hypokalemia, hypercalciuria, hypocitraturia, nephrocalcinosis, and conserved glomerular filtration rate. In some cases, neurosensorial deafness is associated. dRTA is developed during the first months of life and the main manifestations are failure to thrive, vomiting, dehydration, and anorexia.

**Methods:**

Nine unrelated families were studied: seven children, a teenager, and an adult with dRTA. Hearing was preserved in four children. Coding regions of the genes responsible for recessive dRTA were analysed by Sanger sequencing.

**Results:**

Molecular defects were found in the genes *ATP6V1B1* and *ATP6V0A4*. We identified three homozygous variants in *ATP6V1B*: a frameshift mutation (p.Ile386Hisfs*56), a nucleotide substitution in exon 10 (p.Pro346Arg), and a new splicing mutation in intron 5. Three patients were homozygous for one novel (p.Arg743Trp) and one known (p.Asp411Tyr) missense mutations in the *ATP6V0A4* gene. Three patients were compound heterozygous: one proband displayed two novel mutations, the frameshift mutation p.Val52Metfs*25, and a large deletion of exons 18–21; two probands showed the missense mutation p.Asp411Tyr and as a second mutation, p.Arg194Ter and c.1691+2dup, respectively.

**Conclusion:**

*ATP6V0A4* and *ATP6V1B1* genes were involved in recessive dRTA of Mexican families. All *ATP6V1B1* mutations detected were homozygous and all patients developed sensorineural hearing loss (SNHL) early in infancy. *ATP6V0A4* mutations were found in one infant and three children without SNHL, and in one teenager and one adult with SNHL confirming the phenotypic variability in this trait. The mutation p.Asp411Tyr detected in four Mexican families was due to a founder effect. Screening of these mutations could provide a rapid and valuable tool for diagnosis of dRTA in this population.

## Introduction

Hereditary distal renal tubular acidosis (dRTA) results from mutations in genes encoding for three proteins expressed in *α*‐intercalated cells of the collecting duct: the a4 and B1 subunits of the V‐ATPase and the anion exchanger Cl^−^/HCO_3_^−^ (kAE1). Impairment of apical proton secretion or basolateral bicarbonate reabsorption, produced by abnormal function of one of these proteins, is responsible for decreased ammonium (NH_4_^+^) excretion and defect in urine acidification, leading to simultaneous metabolic acidosis, hypokalemia, hypercalciuria, hypocitraturia, and nephrocalcinosis (Escobar Pérez et al. 2013; Gil‐Peña et al. 2014).

*ATP6V1B1* and *ATP6V0A4* genes encode the B1 and a4 subunits of the V‐ATPase, respectively. The V‐ATPase is expressed in the acid secretory *α*‐intercalated cells of the cortical and medullary collecting duct in the kidney and in the epithelial cells of the endolymphatic sac in the cochlea (Dou et al. 2004). Mutations in these genes impair the V‐ATPase proton‐secreting function and produce the autosomal recessive form of dRTA, which can be associated with sensorineural hearing loss (SNHL) (Smith et al. 2000; Stover et al. 2002; Vargas‐Poussou et al. 2006). *ATP6V1B1* mutations are mostly associated with onset SNHL during infancy whereas *ATP6V0A4* mutations are associated with variable hearing phenotypes ranging from early to late onset SNHL (between the ages of 10 and 40 years) (Karet et al. 1999a; Stover et al. 2002; Vargas‐Poussou et al. 2006; Gao et al. 2014).

## Materials and Methods

Six kids were diagnosed with dRTA at the Hospital General del Centro Medico Nacional La Raza IMSS, a teenager at the Hospital Infantil de Mexico Federico Gomez, Mexico city and a child at the Hospital de Especialidades No. 25, Centro Medico Nacional del Noreste, Monterrey (Guerra‐Hernandez et al. 2014). The adult patient was contacted by the web site www.acidosistubular.unam.mx. Clinical diagnosis was supported by the presence of hyperchloremic metabolic acidosis with normal anion gap, hypercalciuria, hypokalemia, nephrocalcinosis, polyuria, and failure to thrive. Hearing was assessed by pure‐tone audiometry.

Informed consent was obtained for blood collection and genetic analysis from patients and children's parents. Patient clinical evolution was followed up from 1 to 3  years. Experiments were performed according to the Declaration of Helsinki and were approved by the hospital′s ethics committee.

Peripheral blood samples were collected on EDTA tubes. Pure DNA was obtained using the QIAamp DNA blood Midi kit (Qiagen) according to the manufacturer′s instructions. The coding exons and intron–exon junctions were amplified with specific primers as previously described (Vargas‐Poussou et al. 2006). Direct sequencing was performed using the dideoxy chain termination method on an automated Perkin Elmer/Applied Biosystems (Foster City, CA).

DNA mutations were identified using Sequencher software by comparison to *ATP6V0A4* and *ATP6V1B1* genes reference sequences: NM_130841 and NM_001692. Each mutation was confirmed by sequencing a second independent PCR product. Missense and splicing mutations were interpreted with Alamut V.2.5.1 software (Interactive Biosoftware, Rouen, France; http://www.interactivebiosoftware.com). Complementary analyses were performed with SIFT ( http://sift.jcvi.org/), PolyPhen‐2 ( http://genetics.bwh.harvard.edu/pph2/index.shtml), Mutpred ( http://mutpred.mutdb.org/about.html), SNPs&Go ( http://snps-and-go.biocomp.unibo.it/snps-and-go/info.htm), and mutation taster ( http://www.mutationtaster.org/).

### Quantitative multiplex PCR of short fluorescent fragments

We adapted the Quantitative multiplex PCR of short fluorescent fragments (QMPSF) method (Houdayer et al. 2004) to detect large deletions or duplications at the *ATP6V0A4* gene. QMPSF consists of a fluorescent multiplex PCR that permits simultaneous amplification of multiple short exonic fragments under semiquantitative conditions. In each QMPSF, a fragment from the hydroxymethylbilane synthase (*HMBS*) gene was amplified as an internal control in each one of three multiplex reactions. After the PCR, the 6FAM‐labeled amplicons are separated by capillary electrophoresis on an ABI Prism 3730XL DNA Analyzer Sequencer (Applied Biosystems). Data were analyzed using GeneMarker Software version 1.85 (Applied Biosystems). For each patient, the mean value of each amplicon was obtained by comparing the peaks between the patient and a reference sample. If this value was below 0.7, the respective exon was defined as deleted; a value between 0.7 and 1.3 was defined as normal. Primers used are shown in Table S1.

### Haplotype analysis

Haplotype analysis was carried out in families harboring the recurrent mutation p.Asp411Tyr for the *ATP6V0A4* gene, to determine whether these families were descended from a common ancestor. Haplotypes were defined by genotyping by direct sequencing three common intragenic single‐base pair polymorphisms (SNPs): rs10258719, rs1026435, and rs3807154, located in exons 2, 15, and 16, respectively.

## Results

### Clinical findings

The main clinical manifestations were dehydration episodes, failure to thrive, malnutrition, and vomiting (Table 1). dRTA is prone to constipation and inability to concentrate the urine due to renal water and potassium losses (Escobar Pérez et al. 2013; Gil‐Peña et al. 2014). Previously, we published the biochemical and clinical findings of five of these patients (Guerra‐Hernandez et al. 2014). Briefly, patients had a clinical history of hyperchloremic metabolic acidosis (venous blood gases with pH 7.2, pCO_2_ 26 mmHg and bicarbonate <14 mEq/L), hypokalemia (potassium 2.2 mEq/L), hypercalciuria, hypocitraturia, and nephrocalcinosis. dRTA is characterized by the loss of the ability to acidify urine by a defect in acid excretion (mainly ammonium) by the collecting tubule. Even when it is not necessary to perform an acidification test for diagnosis of dRTA, impairment of urine acidification was confirmed with the maximum urinary pCO_2_ test using acetazolamide and sodium bicarbonate (Guerra‐Hernández et al. 2015). From the nine patients, only four had bilateral and one unilateral SNHL (Table 1), varying from mild (40 dB) to severe (80 dB).

**Table 1:** Clinical features at diagnosis and current conditions in Mexican patients with recessive dRTA

Patient	Age at diagnosis (months)	BW (kg) length (cm) at birth	Clinical features	Sensorineural hearing loss	Nephrocalcinosis	Current age, years (y), months (m)	Current weight, z score	Current height, z score
I – Female	12	3.1, 49	Vomiting, hypokalemia, dehydration pneumonia, malnutrition	No	Yes	5 y 8 m	−1.99	−2.15
II – Male	4	3.3, 51	Lack of appetite,hypokalemia, vomiting, dehydration, urinary infections	Yes bilateral	Yes	27 y	1.61	0.58
III – Female	2	3.15, 50	Anemia, dehydration, failure to thrive, hypokalemia	Yes unilateral	Yes	13 y	0.61	−1.45
IV – Male	12	3.7, 50	Dehydration, hyperammonemia, hypokalemia, hyperchloremia	No	Yes	4 y 7 m	−1.0	−1.1
V – Female	3	3.2, 51	Vomiting, dehydration, failure to thrive, hypokalemia, diarrhea	No	Yes	4 y 9 m	0.68	−0.07
VI – Male	3	2.4, 47	Dehydration, failure to thrive, hypokalemia	No	Yes	1 y 3 m	−1.6	−1.7
VII – Male	12	2.9, 49	Vomiting, dehydration, failure to thrive, hypokalemia	Yes bilateral	Yes	9 y	1.11	−1.17
VIII – Male	9	3.0, 53	Dehydration, failure to thrive, diarrhea, hypokalemia	Yes bilateral	Yes	5 y 3 m	−2.22	−3.19
IX – Male	41	2.95, 50	Dehydration, failure to thrive, muscle paralysis, delayed motor skills, hypokalemia	Yes bilateral	Yes	4 y 3 m	−0.13	−0.67

### Mutations in the *ATP6V0A4* gene

We identified six different mutations in the *ATP6V0A4* gene in six probands including three novel mutations. The novel mutations comprised a missense mutation (p.Arg743Trp) (patient VI), one small frameshift deletion (p. Val52Metfs*25), and a large deletion of exons 18–21 (patient I). The compound heterozygote (I) with deletion of four nucleotides (154_157del) in exon 3, produced a premature stop codon at Val52Metfs*25. Additionally, direct analysis of exon 3 showed that her mother was heterozygous for this mutation.

Cases III and IV were homozygous for p.Asp411Tyr missense mutation; accordingly, their parents were heterozygous for this mutation.

The adult patient (II) was compound heterozygous for one previously reported nonsense mutation (p.Arg194Ter) and the missense p.Asp411Tyr mutation detected in three other patients.

SNHL was developed during the second decade of life in 2 patients with mutations in *ATP6V0A4* (Table 1).

### Mutations in the *ATP6V1B1* gene

Analysis of the nucleotide sequence of the coding region of the *ATP6V1B1* gene identified mutations in three patients, all of them with SNHL (Table 1).

A homozygous duplication (case VIII) causes a shift in the reading frame from isoleucine 386 introducing a premature stop codon (Stover et al. 2002); his mother was heterozygous for this mutation. One patient (case IX) harbor the homozygous missense mutation p.Pro346Arg previously reported (Karet et al. 1999a) and his parents were heterozygous for this mutation. Case VII was homozygous for a novel splicing mutation (445+1G>C) that likely promotes the exon 5 skipping (Table 2). Although his mother was heterozygous for this intron splicing, his father was not, suggesting the loss of one allele in the father and son. Unfortunately, QMPSF for *ATP6V1B1* gene was not available to analyze this hypothesis.

**Table 2:** Mutations detected in Mexican patients with recessive dRTA

Patient	Gene	Status	Nucleotide^a^	Protein	Exon/Intron	Reference	Nucleotide*	Protein	Exon/Intron	Reference
I	*ATP6V0A4*	Compound heterozygous	c.154_157del	p. Val52Metfs*25	3	This study	c.2011‐?_2523+?del^b^	p.?	18–21	This study
II	*ATP6V0A4*	Compound heterozygous	c.580C>T	p.Arg194Ter	7	Stover et al. (2002)	c.1231G>T	p.Asp411Tyr	12	Pereira et al. 2015
III	*ATP6V0A4*	Homozygous	c.1231G>T	p.Asp411Tyr	12	Barros‐Pereira et al. (2015)	c.1231G>T	p.Asp411Tyr	12	Pereira et al. 2015
IV	*ATP6V0A4*	Homozygous	c.1231G>T	p.Asp411Tyr	12	Barros‐Pereira et al. (2015)	c.1231G>T	p.Asp411Tyr	12	Pereira et al. 2015
V	*ATP6V0A4*	Compound heterozygous	c.1231G>T	p.Asp411Tyr	12	Barros‐Pereira et al. (2015)	c.1691+2dup	p.?	15	Stover et al. 2002
VI	*ATP6V0A4*	Homozygous	c.2227C>T	p.Arg743Trp	19	This study	c.2227C>T	p.Arg743Trp	19	This study
VII	*ATP6V1B1*	Homozygous	c.445+1G>C^c^	p.?	5	This study	c.445+1G>C^c^	p.?	5	This study
VIII	*ATP6V1B1*	Homozygous	c.1155dup	p.Ile386Hisfs*56	12	Stover et al. (2002)	c.1155dup	p.Ile386Hisfs*56	12	Stover et al. 2002
IX	*ATP6V1B1*	Homozygous	c.1037C>G	p.Pro346Arg	10	Karet et al. (1999a, 1999b)	c.1037C>G	p.Pro346Arg	10	Karet et al. 1999a, 1999b

a Nucleotides numbered according to the sequence in GenBank NM_130841 for *ATP6V0A4* and NM_001692 for *ATP6V1B1*. The A of the ATG of the Methionine initiation codon is defined as nucleotide 1. Mutations are described following version 2.0 HGVS recommendations ( http://hgvs.org/mutnomen/).

b Deletion of exons 18 to 21.

c Splice site score is abolished.

?, is the nomenclature used for splicing mutation when the consequence on protein is unknown.

**Figure 1:**
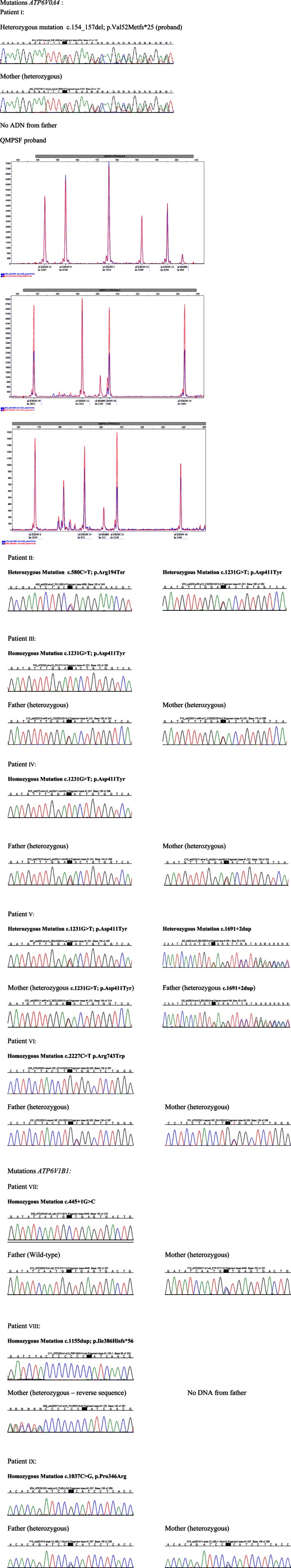
Chromatograms of mutations in the *ATP6V0A44* and *ATP6V1B1* genes detected by direct sequencing and QMPSF. For the QMPSF, each peak represents one analyzed exon and the HMBS internal control. Control samples are shown in red and patients' samples in blue. Proband has QMPSF half doses for exons 18 to 21.

Mutations in the two genes are summarized in Table 2 and the corresponding DNA sequences in Figure 1.

## Discussion

We studied nine probands from independent families who presented clinical features of dRTA. All probands were from nonconsanguineous families. Loss‐of‐function mutations were identified in the two alleles in probands of all families: six probands had mutations in the *ATP6V0A4* gene and three in the *ATP6V1B1* gene.

Mutations in the *ATP6V0A4* gene include 1 novel large deletion, 1 novel frameshift, 1 nonsense, 2 missense, and 1 splicing mutations (Table 2). Most of them, excepting the 2 missense, could result in unstable mRNA or truncated proteins and could be classified as pathogenic variants according to ACMG recommendations (Richards et al. 2015). The two missense mutations, the recently described p.Asp411Tyr and the novel p.Arg743Trp, could be classified as likely pathogenic. Indeed, they affect highly conserved amino acids and are predicted as pathogenic by all the in silico tools; in addition, they have a low frequency in ExAC database. A detailed classification of these variants is given in Table S2. Concerning the large deletion of exons 18–21, to the best of our knowledge, this is the second description of a large rearrangement implicating this gene. Miura et al. described a deletion of exon 15 and a deletion of exons 1–8 (Miura et al. 2013).

*ATP6V0A4* mutations were found in one infant and three children without SNHL, and in one teenager and one adult with SNHL confirming the phenotypic variability in this trait.

**Figure 2:**
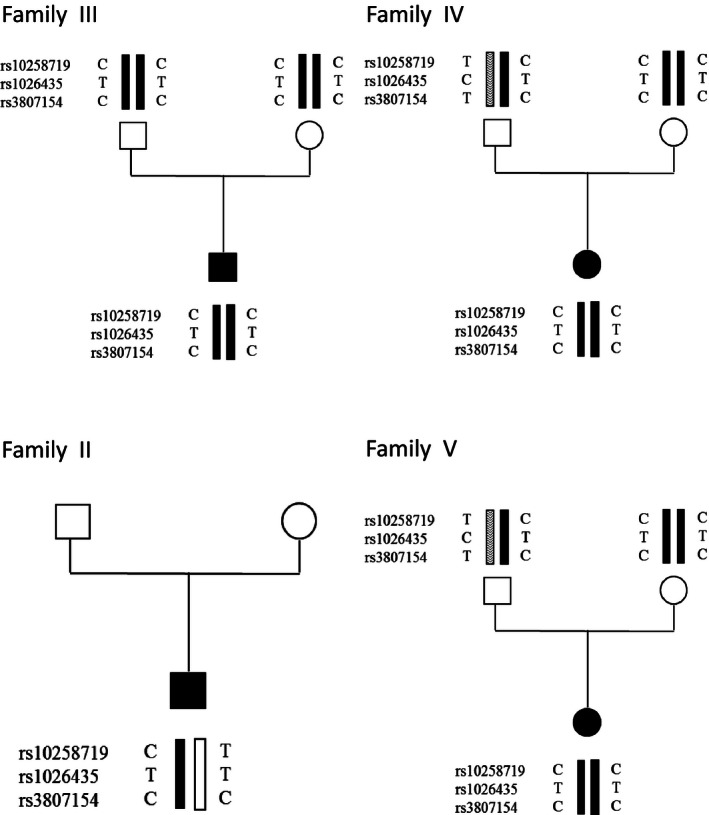
Haplotypes of four Mexican families carrying the p.Asp411Tyr mutation: families III and IV have no history of consanguinity but the mutation was homozygous, as well as the haplotypes. In probands of families II and V the mutation was heterozygous and associated with a second mutation. For patient II, DNA from parents was not available, but he harbors the CTC haplotype in one allele. For patient V, the CTC allele and p.Asp411Tyr mutation were inherited from her mother.

SNP haplotype analysis suggests that mutation p.Asp411Tyr, detected in four Mexican families, is a founder effect. Indeed, patients carrying this mutation shared the same haplotype (CTC) at the disease locus (Fig. 2). Interestingly, this mutation was recently found in one family (a boy and a girl) from Brazil (Pereira et al. 2015).

All *ATP6V1B1* mutations detected were homozygous and all patients developed SNHL early in infancy. Mutations comprise one frameshift that provoked a premature stop codon, one missense mutation, and a novel mutation in intron 5 (a substitution in the first base of splice donor site). The frameshift and splice site mutation could be classified as pathogenic and the known missense mutation as likely pathogenic (Table S2).

*ATP6V0A4* and *ATP6V1B1* genes have been associated with autosomal recessive dRTA of families from Turkey (Karet et al. 1999b), Tunisia (Elhayek et al. 2013) and North Africa (Vargas‐Poussou et al. 2006), mostly from consanguineous marriages, and also from Algeria (Vargas‐Poussou et al. 2006), France (Vargas‐Poussou et al. 2006), Saudi Arabia (Karet et al. 1999b), China (Gao et al. 2014), Greece (Feldman et al. 2006), Italy (Andreucci et al. 2009), Iran (Zeinali et al. 2014), India (Naveen et al. 2015) Pakistan (Vargas‐Poussou et al. 2006), Spain (Gil‐Peña et al. 2007), Serbia (Mohebbi et al. 2013), and Brasil (Pereira et al. 2015).

In conclusion, *ATP6V0A4* and *ATP6V1B1* genes are involved in recessive dRTA of Mexican families. This study constitutes the first genetic analysis of Mexican families with autosomal recessive dRTA. These data show that analysis of these genes is a good predictor for future screenings and molecular diagnostic of dRTA in this population.

## Conflict of interest

The authors declare no conflicts of interest.

## Supporting information


**Table S1.** Primers used for QMPSF
**Table S2**. Classification of the variants detected in Mexican families with distal renal tubular acidosis
