# Supplementary material for: Mutations in ATP6V1B1 and ATP6V0A4 genes cause recessive distal renal tubular acidosis in Mexican families
Source: Mol Genet Genomic Med. 2016 Feb 14;4(3):303–11. doi: 10.1002/mgg3.205 (PMC4867564; doi:10.1002/mgg3.205)
Supplement: Supplementary file 1 — Table S1. Primers used for QMPSF Table S2. Classification of the variants detected in Mexican families with distal renal tubular acidosis [file MGG3-4-303-s001.docx]

Supplementary table 1. Primers used for QMPSF

| **Multiplex A** | Forward 5' - 3' | Reverse 5' - 3' | Amplicon size |
| --- | --- | --- | --- |
| **Exon 2** | CGTTAGATAG-TTCACCCTAAGGTTTTCACACA | GATAGGGTTA-ACCCCTGCCTTCCTCTTATC | 241 |
| **Exon 3** | CGTTAGATAG-GAAATCTATTGGCAGCTGTT | GATAGGGTTA-AACTATTAACCCCCTTGCCA | 201 |
| **Exon 5** | CGTTAGATAG-GTTGTGGGTAATGTATTTCAG | GATAGGGTTA-AGCACTACAATTATTCCACC | 364 |
| **Exon 7** | CGTTAGATAG-CCCTTCCTTTCATGGTGTGT | GATAGGGTTA-CTTCTGCTGAGGGCTATGG | 282 |
| **Exon 11** | CGTTAGATAG-AGGAGCTGGGACTGTGAC | GATAGGGTTA-CTCAGGGGTCCATCTTAC | 303 |
| **Exon 15** | CGTTAGATAG-TGTAGCCCTCAACTCCAGTA | GATAGGGTTA-CCAGATGCCCAGGGAAGTAC | 326 |
| **Exon 18** | CGTTAGATAG-GAGGGCTTAATGAGATAGTG | GATAGGGTTA-TGAGACCTTCACAGCTTC | 418 |
| **Multiplex B** |  |  |  |
| **Exon 4** | CGTTAGATAG-GCTTGACCTCATTTCTTAGTTA | GATAGGGTTA-TGGGATTTTCCTGGGGCTGT | 333 |
| **Exon 6** | CGTTAGATAG-GTGTAGGTGCCTTTTGAGTT | GATAGGGTTA-TTCACCCGTTCATTCACTCA | 236 |
| **Exon 9** | CGTTAGATAG-AGAAAAGGGGATGCAGAATG | GATAGGGTTA-CATAGCCAGCATTCCAGCCA | 283 |
| **Exon 10** | CGTTAGATAG-GTCAAAGGAGCCTGCCAGAG | GATAGGGTTA-AGCAATCCTACCACCAAGT | 447 |
| **Exon 14** | CGTTAGATAG-CAAGTTCGTGACTGGTGGTA | GATAGGGTTA-GAAAATGGGGCTGGGTAGAA | 377 |
| **Exon 20** | CGTTAGATAG-TCCTAGGCAACAGAGTGAGA | GATAGGGTTA-GTCACATACAGCTCACGATC | 401 |
| **Exon 21** | CGTTAGATAG-CTTTGTGCTTGATTTTACG | GATAGGGTTA- CAGGTGAGCCAAGAACAAC | 321 |
| **Multiplex C** |  |  |  |
| **Exon 8** | CGTTAGATAG-TGAGCTATGAACTTAGATGA | GATAGGGTTA-GTTATTCTAAAGCCTTACTG | 341 |
| **Exon 12** | CGTTAGATAG-CGCATGCAAATCGTGGAG | GATAGGGTTA-ATCCAAAGGAATTACCTTTG | 401 |
| **Exon 13** | CGTTAGATAG-GTCTGGATGATTGATGTCTG | GATAGGGTTA-CCTGGCCTATTTGAATTTTC | 432 |
| **Exon 16** | CGTTAGATAG-TGCCGTTGTTTGCCAGTAGT | GATAGGGTTA-TGGGTGACAGAGCAAGACTC | 458 |
| **Exon 17** | CGTTAGATAG-TGGATTCATTGAAATGCTTT | GATAGGGTTA-CCCAGGACGATTCTCTCTAA | 328 |
| **Exon 19** | CGTTAGATAG-CAGCAAAGCCCATGTCTTAT | GATAGGGTTA-AATAGCTCGGAGAAGTCAC | 313 |
| **HMBS** | CGTTAGATAG-TAGACGGCTCAGATAGCATACAAG | GATAGGGTTA-ATGCCTACCAACTGTGGGTCA | 187 |

Supplementary Table 2. Classification of the variants detected in Mexican families with distal renal tubular acidosis

| **Case** | **Gene** | **Nucleotide variant** | **Aminoacid**  **change** | **Coding effect** | **MAF***  **(ExAC database)** | **SIFT score** | **Grantham**  **distance** | **PolyPhen-2 score** | **MutPred** | **MutationTaster** | **Splice site** | **HGMD ID and reference** | **Classification** |
| --- | --- | --- | --- | --- | --- | --- | --- | --- | --- | --- | --- | --- | --- |
| I | *ATP6V0A4* | c.154_157del,  c.2011-  ?_2523+?del | p. (Val52Metfs*25)  p.? | Frameshift  Large deletion | Total 1/121184  Latino 0/11564  Absent | -  - | -  - | -  - | -  - | -  - | -  - | -  - | Pathogenic  Pathogenic |
| II | *ATP6V0A4* | c.580C>T  c.1231G>T | p.(Arg194Ter)  p.(Asp411Tyr) | Nonsense  Missense | Absent  Total 8/121062  Latino 7/11514 | -  0 | -  160 | -  1 | -  0.94 | -  1 | - | CM023019/Stover et al. 2002  Barros-Pereira et al. 2015 | Pathogenic  Likely pathogenic |
| III, IV | *ATP6V0A4* | c.1231G>T | p.(Asp411Tyr) | Missense | Total 8/121062  Latino 7/11514 | 0 | 160 | 1 | 0.94 | 1 |  | Barros-Pereira et al. 2015 | Likely pathogenic |
| V | *ATP6V0A4* | c.1231G>T  c.1691+2dup | p.(Asp411Tyr)  p.? | Missense  Splicing | Total 8/121062  Latino 7/11514  Total 1/121172  Latino 1/11528 | 0 | 160 | 1 | 0.94 | 1 | MaxEnt: -100.0%  NNSPLICE: -99.6%  HSF: -30.5% | Barros-Pereira et al. 2015  CI023292/Stover et al. 2002 | Likely pathogenic  Pathogenic |
| VI | *ATP6V0A4* | c.2227C>T | p.(Arg743Trp) | Missense | Total 2/121314  Latino 2/11578 | 0 | 101 | 1 | 0.93 | 1 |  | - | Likely pathogenic |
| VII | *ATP6V1B1* | c.445+1G>C | p.? | Splicing | Absent |  |  |  |  |  | MaxEnt: -100.0%  NNSPLICE: -100.0%  HSF: -100.0% | - | Pathogenic |
| VIII | *ATP6V1B1* | c.1155dup | p.(Ile386Hisfs*56) | Frameshift | Absent |  |  |  |  |  |  | CD991636/ Karet et al. 1999 | Pathogenic |
| IX | *ATP6V1B1* | c.1037C>G | p.Pro346Arg | Missense | Total 2/120872  Latino 1/11564 | 0 | 103 | 1 | 0.91 | 1 |  | CM990272/ Karet et al. 1999 | Likely pathogenic |

The table shows the variants with classification following the ACMG recommendations, including detailed information of the parameters in the *in silico* analysis.

MAF: Minor allele frequency in ExAC database (http://exac.broadinstitute.org/). For SIFT: a change is predicted to be deleterious if the score <0.05. For PolyPhen-2 the score range is from 0.0 (benign) to 1 (damaging). For MutPred: achange is predicted to be deleterious if the general score g > 0.75. For MutationTaster: a change is predicted to be disease causing if the score p >0.5. For SNPs&GO: a change is predicted to be disease causing if the reliability index (ri)>5. Splice site: predicted changes at donor site with 3 different algorithms. HGMD ID: Identification number in the Human Gene Mutation Database.
